# Practical aspects of the application of helical tomotherapy for craniospinal irradiation

**DOI:** 10.1038/s41598-021-85574-y

**Published:** 2021-03-17

**Authors:** Joongyo Lee, Euidam Kim, Nalee Kim, Hwa Kyung Byun, Chang-Ok Suh, Yoonsun Chung, Hong In Yoon

**Affiliations:** 1 Department of Radiation Oncology, Yonsei Cancer Center, Yonsei University Health System, Yonsei University College of Medicine, 50-1 Yonsei-ro, Seodaemun-gu, Seoul, 03722 Republic of Korea; 2 Department of Nuclear Engineering, Hanyang University, 222 Wangsimni-ro, Seongdong-gu, Seoul, 04763 Republic of Korea; 3 Department of Radiation Oncology, Samsung Medical Center, Sungkyunkwan University School of Medicine, Seoul, Republic of Korea; 4 Department of Radiation Oncology, Bundang CHA Medical Center, CHA University, Gyeonggi-do, Republic of Korea

**Keywords:** Radiotherapy, Outcomes research

## Abstract

We investigated the practical aspects of the application of craniospinal irradiation using helical tomotherapy (HT-CSI) by evaluating interfractional setup errors and intrafractional movement during each treatment in 83 patients undergoing HT-CSI between January 2014 and December 2018. Interfractional setup errors in each axis (mediolateral; ML, craniocaudal; CC, and anteroposterior; AP) were assessed as differences between pre-treatment megavoltage computed tomography (MVCT) images scanned (zygomatic arch to the C4 spine) and planning CT images. Intrafractional movements were evaluated as the difference between pre-treatment and post-treatment MVCT (T12–L4 spine) images at each fraction. Median interfractional setup error was acceptable in every axis (ML: 1.6 mm, CC: 1.9 mm, AP: 3.1 mm). Seven patients (8.4%) experienced significant intrafractional displacement from 1 to 10 fractions (0.34% for ML, 0.74% for CC, 1.21% for AP). Weight loss grade 1+ during treatment (*p* = 0.016) was an independent risk factor for significant intrafractional displacement. The risk factor for significant intrafractional movement in pediatric patients was weight loss grade 1+ (*p* = 0.020), while there was no factor in adults. HT-CSI could be a feasible treatment modality with acceptable setup verification. Inter- and intrafractional errors were acceptable; paying attention to weight loss during treatment is necessary, especially in pediatric patients.

## Introduction

Craniospinal irradiation (CSI) is often needed in patients with brain tumors who are at risk of dispersion through the cerebrospinal fluid^1–4^. CSI is a complex radiotherapy (RT) technique used for the cranium and spinal cord, with the movement of junctions along the lateral brain and spinal field of the patient.

Although three-dimensional conformal RT (3D-CRT) has been the most common and useful technique for CSI, CSI using 3D-CRT still has many limitations such as problems related to multiple isocenters, need for junction movement during treatment, and dose inhomogeneity at the beam junctions^5–7^. Additionally, large areas of the organs at risk (OARs) near the target can be irradiated due to the low conformity of 3D-CRT compared to those of the latest RT techniques^6,8–10^. These drawbacks of the 3D-CRT technique are highlighted by the fact that CSI is generally used for pediatric patients because they are known to have more severe side effects such as endocrine and fertility dysfunction, growth and musculoskeletal abnormalities, neurobehavioral deficits, and secondary malignancies due to unnecessary irradiation to OARs^4,11–14^.

After 3D-CRT, many RT technologies have been developed, such as intensity-modulated RT including helical tomotherapy (HT), volumetric modulated arc therapy, and particle beam therapy, to overcome the limitations of 3D-CRT and to make RT technique more accurate and precise^15–23^. By using HT for CSI, treatment of extended volume along the craniocaudal direction and more homogeneous dose distribution is possible without any junction-related problems during treatment, owing to continuous helical delivery of the intensity-modulated fan beam in HT^24^. In addition, treatment in a more comfortable position to the patient (both prone or supine) is possible, and daily patient position can be verified using megavoltage computed tomography (MVCT) at every treatment fraction^4,22,24–26^. However, there are also some potential drawbacks of HT: poor image quality and setup uncertainty owing to the use of MVCT. In HT, problem of poor image quality has been raised as a one of the major limitations of MVCT imaging. Moreover, HT also involves setup uncertainty due to the relatively short range of MVCT compared to the long range of the treatment field in the craniocaudal direction required for CSI using HT (HT-CSI)^16,27,28^. Among these drawbacks, in terms of the image quality, Forrest et al. and Meeks et al. reported that MVCT can provide sufficient image quality for tumor identification and setup verification, despite its low-contrast resolution for soft tissues^29,30^. However, questions remain about setup uncertainty before or during treatment.

In this study, the clinical feasibility of HT-CSI was demonstrated by investigating the accuracy of setup in interfractional and intrafractional aspects, focusing on the median setup error and existence of significant movement during treatment in both groups, and identifying clinically significant risk factors for intrafractional movement.

## Results

### Patient characteristics

Median patient age at HT-CSI was 20.9 years (range, 12.1–31.2 years); 48.2% of patients were pediatric patients (patients under the age of 20 years). The most common pathology was germ cell tumor for pediatric patients (15 patients, 37.5%) and glioblastoma for adult patients (10 patients, 23.3%). The baseline characteristics of patients are summarized in Table 1.

**Table 1:** Patient and treatment characteristics.

Characteristic	N	%
**Age (years, median [range])**	21 (2–74)	
< 20 years (pediatric)	40	48.2
≥ 20 years (adult)	43	51.8
**Histology–Pediatric**		
Germ cell tumor	15	37.5
Medulloblastoma	14	35
Miscellaneous	11	27.5
**Histology–Adult**		
Glioblastoma	10	23.3
Leptomeningeal carcinomatosis	7	16.3
Germ cell tumor	4	9.3
Miscellaneous	22	51.2
**Sex**		
Male	54	55.6
Female	29	44.4
Height (cm, median [range])	165 (88–192)	
Body-mass index (kg/m^2^, median [range])	20.4 (10.9–28.4)	
Weight loss grade ≥ 1*	13	15.7
Nausea grade ≥ 2*	20	24.1
Total CSI dose (Gy, median [range])	36.0 (12.0–45.0)	
Total CSI fraction number (fractions, median [range])	20 (8–30)	
Fractional CSI dose (Gy, median [range])	1.5 (1.2–3.0)	
**CSI field**		
Brain-Sacrum	76	91.6
Posterior fossa-Sacrum	2	2.4
C1 spine-Sacrum	5	6.0
Beam on time (seconds, median [range])	538.1 (310.6–964.8)	
Sedation during treatment	7	8.4
Concurrent chemotherapy	29	34.9
Overall CSI treatment time (days, median [range])	32 (11–108)	
**Medically-indicated treatment interruptions**	28	33.7
Days (median [range])	5 (1–58)	
Adaptive during CSI	3	3.6

*CSI* craniospinal irradiation; *Gy* gray.

*Based on common terminology criteria for adverse events version 5.0.

With a median body mass index (BMI) of 20.4 kg/m^2^, 26 patients were categorized as underweight (BMI < 18.5 kg/m^2^), 50 were categorized to have normal weight (BMI 18.5–25 kg/m^2^), and 7 were categorized to have overweight (BMI 25–30 kg/m^2^) according to the World Health Organization criteria^31^. Weight loss and nausea were evaluated according to Criteria for Adverse Events (CTCAE), version 5.0. There were 13 patients (15.7%) who experienced weight loss grade 1+ (≥ 5% from baseline) and 20 patients (24.1%) with nausea grade 2+ (outpatient intravenous hydration; medical intervention indicated) during HT-CSI.

### Treatment characteristics

Treatment characteristics are listed in Table 1. The median total HT-CSI dose for all patients was 36.0 Gy (range, 12.0–45.0 Gy), with a median fractional dose of 1.5 Gy (range, 1.2–3.0 Gy). Most patients (76 patients, 91.6%) received HT-CSI from the whole brain to the sacral level, 5 (6.0%) received irradiation from the C1 spine level to the sacral level, and 2 (2.4%) received irradiation from the posterior fossa to the sacral level. The median beam on time was 538.1 s (range, 310.6–964.8 s). Concurrent chemotherapy was administered to 29 patients (34.9%).

The median overall treatment time for HT-CSI was 32 days (range, 11–108 days), and in 28 patients (33.7%), treatment was interrupted mainly due to thrombocytopenia. Of the 28 patients, adaptive treatment planning was performed on newly taken simulation computed tomography (CT) images in 3 patients due to long-term treatment interruption.

### Interfractional setup errors

**Figure 1:**
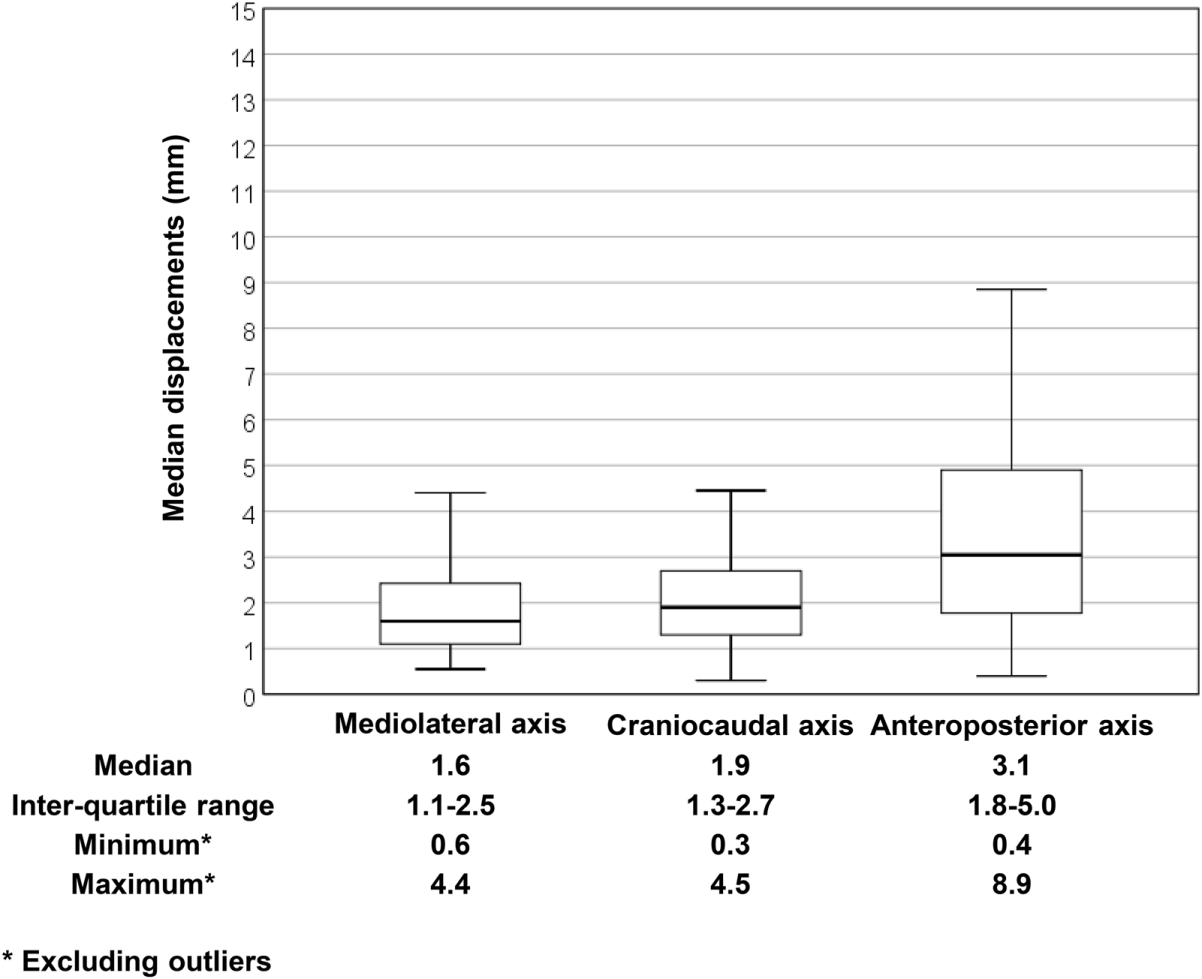
Distribution of median interfractional displacements in the three major axes. The top end and the bottom end of the error bar are the maximum and minimum value excluding outliers of the interfractional displacements of three axes, respectively. The outliers were defined as larger (smaller) values than the upper (lower) boundary, which were defined as 1.5 IQR above (below) 75th (25th) percentile. Figure created in IBM SPSS, version 23.0 (https://www.ibm.com/analytics/spss-statistics-sofware).

The interfractional setup errors obtained from each axis are summarized in Fig. 1. Median values of median shifts in the mediolateral (ML), craniocaudal (CC), and anteroposterior (AP) axes were 1.6 mm (inter-quartile range [IQR] 1.1–2.5 mm), 1.9 mm (IQR 1.3–2.7 mm), and 3.1 mm (IQR 1.8–5.0 mm), respectively. The systematic errors (Σ) in the ML, CC, and AP axes were 1.17 mm, 2.83 mm, and 1.75 mm, respectively. The random errors (σ) in the corresponding directions were 1.37 mm, 2.52 mm, and 1.95 mm, respectively.

### Intrafractional movement

For intrafractional movement, median values of median shifts in the ML, CC, and AP axes were 2.1 mm (IQR 1.5–3.2 mm), 1.9 mm (IQR 1.0–2.7 mm), and 2.3 mm (IQR 1.4–3.7 mm), respectively. Of the total 1,483 fractions, significant intrafractional displacement occurred 5 times (0.34%) in the ML direction, 11 times (0.74%) in the CC direction, and 18 times (1.21%) in the AP direction. A total of 7 patients showed significant intrafractional displacement in one or more axes at least once during the treatment course. Of the 7 patients, 1 showed significant intrafractional displacement in all axes; 3 patients, in two of the three axes; and 3 patients, in one axis (one for each of the ML, CC, and AP axes). Median intrafractional movement in the ML, CC, and AP axes for the 7 patients was 3.3 mm, 3.4 mm, and 5.8 mm, respectively. The number of significant intrafractional displacements in each patient ranged from 1 to 10. One patient receiving 16 fractions of HT-CSI experienced 10 significant displacements and showed nausea grade 2+ and weight loss of 3.7% compared to the baseline weight.

Multivariate analysis revealed that only weight loss grade 1+ was a risk factor for significant intrafractional displacement (odds ratio, 8.10, 95% CI: 1.47–44.58, *p* = 0.016, Table 2).

**Table 2:** Risk factors for significant intrafractional movement.

	Univariate analysis		Multivariate analysis	
	OR (95% CI)	*p* value	OR (95% CI)	*p* value
Age (< 20 years vs. ≥ 20 years)	0.34 (0.06–1.87)	0.216		
BMI*	0.87 (0.71–1.07)	0.191		
Total CSI dose*	1.00 (1.00–1.00)	0.957		
Beam on time (< 8 min vs. ≥ 8 min)	1.63 (0.30–8.95)	0.574		
Concurrent chemotherapy (No vs. Yes)	5.42 (0.98–29.94)	0.053	4.24 (0.71–25.43)	0.114
Overall CSI treatment time (< 32 days vs. ≥ 32 days)	0.36 (0.07–1.97)	0.239		
Sedation during treatment (No vs. Yes)	0.00 (0.00–0.00)	0.999		
Medically-indicated treatment interruptions (No vs. Yes)	0.76 (0.14–4.24)	0.763		
Weight loss (Grade < 1 vs. Grade ≥ 1)	9.93 (1.91–51.72)	0.006	8.10 (1.47–44.58)	0.016
Nausea (Grade < 2 vs. Grade ≥ 2)	5.00 (1.01–24.65)	0.048		

*OR* odds ratio; *CI* confidence interval; *BMI* body-mass index; *CSI* craniospinal irradiation.

*BMI and total CSI dose was treated as a continuous variable.

The foreparts of the parentheses were set as the reference groups in the multivariable analysis.

### Subgroup analysis of pediatric and adult patients

Pediatric and adult patients were defined as patients below and above the age of 20 years, and the patient and treatment characteristics of each group are listed in Supplementary Table S1.

For 40 pediatric patients, median interfractional shifts in the ML, CC, and AP axes were 1.6 mm (IQR 1.1–2.4 mm), 1.9 mm (IQR 1.3–2.7 mm), and 3.4 mm (IQR 1.8–5.1 mm), respectively. Of the total 7 patients with significant intrafractional displacement in one or more axes at least once during the treatment period, 5 were pediatric patients. In both univariate and multivariate analyses, weight loss grade 1+ was found to be a risk factor for significant intrafractional displacement (odds ratio, 11.63, 95% CI: 1.47–92.14, p = 0.020, Table 3).

**Table 3:** Risk factors for significant intrafractional movement in pediatric patients.

	Univariate analysis		Multivariate analysis	
	OR (95% CI)	*p* value	OR (95% CI)	*p* value
BMI*	0.83 (0.64–1.08)	0.162		
Total CSI dose*	1.00 (1.00–1.00)	0.156		
Beam on time (< 8 min vs. ≥ 8 min)	1.42 (0.21–9.55)	0.721		
Concurrent chemotherapy (No vs. Yes)	6.21 × 10^8^ (0.00–0.00)	0.998		
Overall CSI treatment time (< 24 days vs. ≥ 24 days)	0.71 (0.11–4.76)	0.721		
Sedation during treatment (No vs. Yes)	0.00 (0.00–0.00)	0.999		
Medically-indicated treatment interruptions (No vs. Yes)	0.42 (0.04–4.20)	0.463		
Weight loss (Grade < 1 vs. Grade ≥ 1)	11.63 (1.47–92.14)	0.020	11.63 (1.47–92.14)	0.020
Nausea (Grade < 2 vs. Grade ≥ 2)	1.93 (0.28–13.44)	0.509		

*OR* odds ratio; *CI* confidence interval; *BMI* body-mass index; *CSI* craniospinal irradiation.

*BMI and total CSI dose was treated as a continuous variable.

The foreparts of the parentheses were set as the reference groups in the multivariable analysis.

In the 43 adult patients, median interfractional shifts in the ML, CC, and AP axes were 1.7 mm (IQR 1.1–2.6 mm), 1.9 mm (IQR 1.2–3.0 mm), and 2.9 mm (IQR 1.8–4.5 mm), respectively. Two adult patients showed significant intrafractional displacement in one or more axes at least once during the treatment period. Of the two patients, one patient was 20 years old, the youngest among adult patients. No factor was significantly associated with significant intrafractional displacement in both univariate and multivariate analyses (Table 4).

**Table 4:** Risk factors for significant intrafractional movement in adult patients.

	Univariate analysis		Multivariate analysis	
	OR (95% CI)	*p* value	OR (95% CI)	*p* value
BMI*	1.54 (0.74–3.23)	0.251		
Total CSI dose*	0.99 (0.99–1.00)	0.228	1.00 (1.00–1.00)	0.115
Beam on time (< 8 min vs. ≥ 8 min)	1.15 × 10^8^ (0.00–0.00)	0.999		
Concurrent chemotherapy (No vs. Yes)	0.00 (0.00–0.00)	0.999		
Overall CSI treatment time (< 35 days vs. ≥ 35 days)	0.95 (0.06–16.28)	0.973		
Sedation during treatment (No vs. Yes)	0.00 (0.00–0.00)	0.999		
Medically-indicated treatment interruptions (No vs. Yes)	2.15 (0.13–37.19)	0.598		
Weight loss (Grade < 1 vs. Grade ≥ 1)	7.20 (0.39–134.22)	0.186	38.13 (0.41–3538.38)	0.115
Nausea (Grade < 2 vs. Grade ≥ 2)	4.62 × 10^2^ (0.00–0.00)	0.998		

*OR* odds ratio; *CI* confidence interval; *BMI* body-mass index; *CSI* craniospinal irradiation.

*BMI and total CSI dose was treated as a continuous variable.

The foreparts of the parentheses were set as the reference groups in the multivariable analysis.

## Discussion

In this study, to demonstrate the feasibility of CSI using HT, we evaluated the accuracy of setup in interfractional and intrafractional movements based on setup errors and patient movement during treatment, respectively. In addition, we identified the risk factors for intrafractional movement using clinically/statistically significant features.

In terms of interfractional setup errors, the median of the interfractional setup errors were less than or equal to 3.1 mm in the ML, CC, and AP axes. The maximum systematic and random errors along the ML, CC, and AP directions were 2.83 mm and 2.52 mm, respectively, which were found to correspond to the results of previous studies by Al-Wassia et al. and Thondykandy et al. (range, 1.1–2.7 mm and 1.9–2.2 mm, respectively)^32,33^.

In terms of intrafractional movement, the overall patient group showed less than or equal to 2.3 mm of median intrafractional movement in every direction. Median intrafractional movements in both pediatric and adult patients were not significantly different. Multivariate logistic regression analyses in all patients showed that weight loss grade 1+ was a risk factor for significant intrafractional movement. The results of the subgroup analysis showed that weight loss grade 1+ was a risk factor for significant intrafractional movement in the pediatric patient group, but no risk factor was reported in the adult patient group.

Several studies have reported that weight loss affects interfractional set up errors^34,35^, but there is no report on intrafractional movement. The association between weight loss and intrafractional movement in our study can be discussed in terms of the following two aspects: first, due to toxicity or nausea induced by a high radiation dose during RT, the patient’s appetite deteriorates, leading to a poor condition; therefore, the weight decreases with progression of the treatment. Second, the patient will not be able to maintain a constant posture during treatment because of irresistible movement caused by nausea or dizziness due to RT or chemotherapy; thus, significant intrafractional movement might be induced.

There are three major limitations to this study. First, although HT can technically scan up to 300 slices, a long-range MVCT scan has not been used in this study, which can be thought of as a limitation of this study. However, in the case of long-range MVCT scan, as the scanning time increases due to its range of long-range MVCT scan, the imaging dose delivered to the patient and the possibility of additional movement of the patient can be increased. Therefore, the long-range MVCT scan was not used when registering the HT-CSI in this study. Second, in HT-CSI protocol of Yonsei Cancer Center, the range of pre-treatment MVCT is from the zygomatic arch to the C4 spine. With the registration of this pre-treatment MVCT and planning CT, it is confirmed that the patient's position before the treatment is same as that of the planning CT under the assumption that the position of the whole body can be confirmed with the pre-treatment MVCT. Through this assumption, is possible to reduce the image dose and treatment time, but it is not certain that the range of pre-treatment MVCT and the whole body are perfectly synchronized in the same state with the planning CT. To minimize this uncertainty, a line is drawn along the midline of the patient’s body during the simulation. This line is aligned with the room laser before each treatment, and pre-treatment MVCT is taken in this state. With this line and laser, the spinal position of the patient in planning CT is synchronized with the cranial position in pre-treatment (verified with pre-treatment MVCT), and can be synchronized with the spinal position in pre-treatment, which can be the basis of the assumptions: position of the whole body can be confirmed with the pre-treatment MVCT. Additionally, considering this assumption and following uncertainty, the larger margin of the spine was applied than that of the brain. However, despite these protocols, there is still a question of perfect synchronization between spinal position in pre-treatment and planning CT cannot be guaranteed. Therefore, further study regarding the effect of this synchronization and uncertainty would be needed to compensate this limitation. Third, in the interfractional setup error analysis, the overestimation of interfractional setup error may be induced. For example, in case of patients who had an incorrect initial positioning during simulation but did not re-simulate, it can be evaluated that these patients represent a large interfractional error despite they did not show the significantly different location in each fraction. The fact that these patients have large interfractional error but did not have clinically significant conditions due to little difference between each fraction can indicate that using the median difference between the patient location in each fraction and planning as an interfractional setup error can overestimate the uncertainty in the setup. These patients who repetitively showed large setup errors were included in the pediatric group; the patient needs to relax and adjust to a comfortable and repeatable position during simulation, and this would be difficult considering that pediatric patients could be particularly nervous during the whole RT procedure.

Additionally, when it comes to planning CT images, 3 mm or smaller CT slice thickness would be more appropriate compared with our setup errors (around 3 mm). However, in case of planning CT for CSI, there could be possible hazard of additional imaging dose to the patient and the movement of patient due to the required additional time with smaller slice thickness compared to current 3 or 5 mm.

Despite these limitations, our research has three strengths. First, our study analyzed 1,483 fractions in a total of 83 patients who received CSI, which is the largest number of patients analyzed with respect to CSI studies using HT. Second, while most of the HT-CSI studies analyzed either interfractional setup errors or intrafractional movement, our study is the only one to analyze and report both error and movement. Finally, to date, no attempt has been made to incorporate clinical factors into HT-CSI-related set up errors, and this is the first study to closely analyze clinical factors related to intrafractional movement.

In conclusion, our findings have demonstrated that HT-CSI would be clinically feasible in terms of interfractional setup errors and intrafractional movements. The effect of difference in scan range of pre- and post- MVCT to setup uncertainty should be analyzed to increase the accuracy of the interfractional setup error. To minimize intrafractional movements in HT-CSI, treatment can be carried out by paying attention to weight loss using best supportive care during CSI. Particularly, for pediatric patients with severe weight loss, delicate monitoring during treatment setup and conservative management are required to maintain the required weight.

## Materials and methods

### Patient selection

CSI using helical TomoTherapy (Accuray Inc., Sunnyvale, CA, USA) has been utilized in Yonsei Cancer Center from 2014. Patients who received HT-CSI between January 2014 and December 2018 were screened (n = 99). Patients were excluded from the study if they met one of the following criteria: (1) they received HT-CSI combined with 3D-CRT (n = 3), (2) they could not complete RT (n = 10), and (3) they did not have any record for translational displacements obtained through MVCT (n = 3). Finally, 83 patients were included in our cohort. Among 83 patients, 21, 42, 19 and 1 patients received HT-CSI using Hi-Art, Tomo-HD, Tomo-HDA and Radixact, respectively.

This study was approved by the Severance Hospital institutional review board (No. 4–2020-0046), and the requirement for informed consent was waived because of the retrospective nature of this study. All methods were carried out in accordance with relevant guidelines and regulations.

### Simulation, treatment planning, and treatment setup

**Figure 2:**
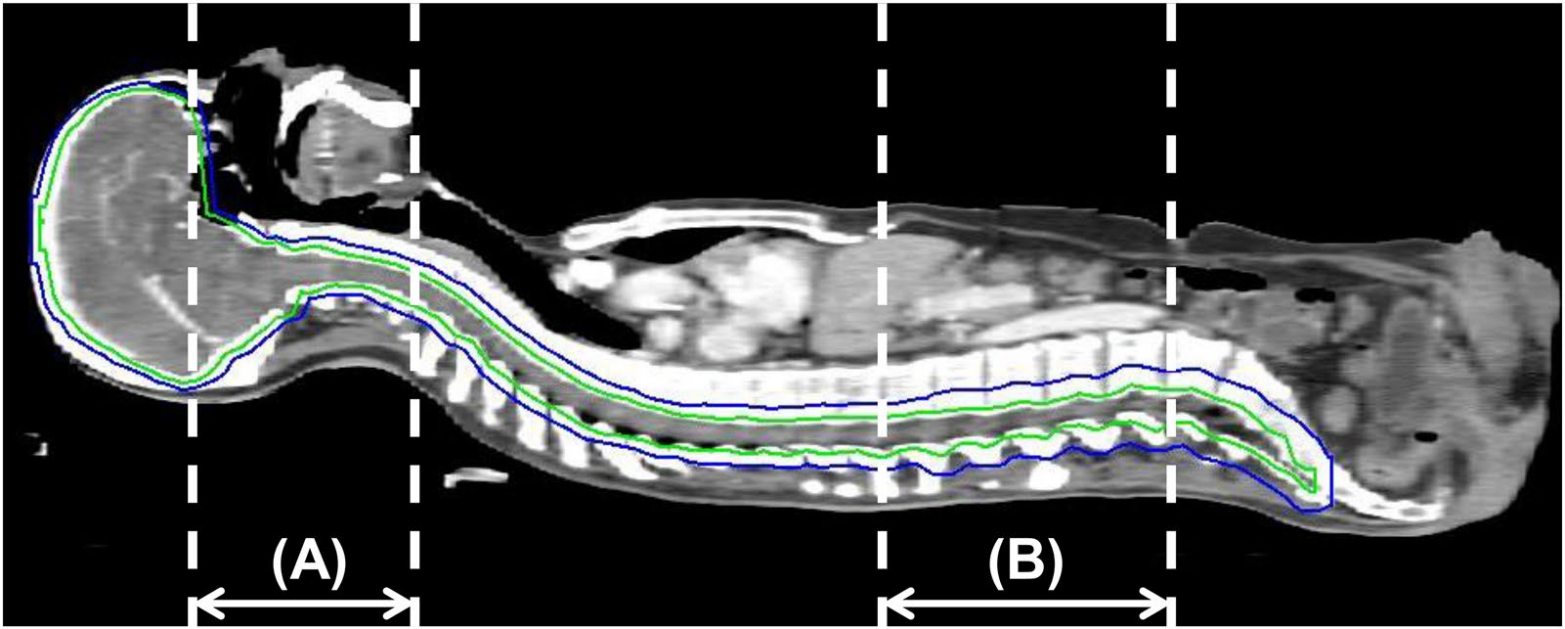
Target delineation for craniospinal irradiation (green line, clinical target volume [CTV]; blue line, planning target volume [PTV]) (**A**) Range of daily pre-treatment megavoltage computed tomography (MVCT). (**B**) Range of daily post-treatment MVCT.

For posture fixation in patients during HT-CSI, the head and neck were immobilized with a thermoplastic mask in the supine position, and the entire body was immobilized with a Vac-Lok cushion (BlueBAG, Elekta, Stockholm, Sweden). A long line is drawn along the midline of the patient’s body to align the spine of the patient. Planning CT images were acquired with 3- or 5-mm slice thickness and with intravenous contrast. The clinical target volume was defined as a 3-mm margin from the whole brain and spinal canal, inferiorly to the end of the dural sac. The planning target volume (PTV) was defined as the expansion of the clinical target volume with a 3-mm margin for the brain, 5-mm margin for the C1–T7 spine level, 7-mm margin for the T8–T12 spine level, and 10-mm margin for the L1 spine–sacral level in all directions (Fig. 2).

For each daily treatment, the patient's posture was fixed with the devices described above as the first step, and then the treatment room laser was aligned to the marks of the Vac-Lok and the thermoplastic mask and to the midline of the patient’s body. Afterwards, an MVCT scan in the coarse mode was obtained from the zygomatic arch to the C4 spine level (Fig. 2A). The HT console software program can automatically register planning CT images using MVCT and determine the direction of PTV displacement with regard to the isocenter. After automatic registration, radiation therapists slightly adjusted the registration manually if necessary. After adjustment, the couch was moved in the ML, CC, and AP directions to the correct setting. In terms of the possible rotational error, every setup was under rotational correction by re-setup when the rotation of pitch/yaw direction more than 1 degree or roll direction more than 2 degrees occurs. Then, pitch and yaw error which were lower than 1 degree were neglected, and the roll correction was registered automatically by HT.

After each fractional treatment was delivered, a post-treatment MVCT scan in the coarse mode was acquired (from the T12 spine to L4 spine) (Fig. 2B). Additionally, post-treatment MVCT images were compared with planning CT images, and the movement in each axis during treatment was determined.

### Analysis of interfractional and intrafractional errors

For the analyses of interfractional and intrafractional errors, both pre- and post-treatment MVCT images of a total of 1,483 fractions in 83 patients were analyzed.

Interfractional setup errors are measurements used for quantifying the day-to-day difference, which are obtained from the translational displacement values in each axis after scanning pre-treatment MVCT. The median displacement in each axis was obtained during the course of the treatment for each patient. To describe the dispersion of the interfractional setup error, the interquartile range, which means the middle 50% of values sorted from lowest to highest, was used. Moreover, systematic error (Σ) and random error (σ) were calculated, wherein systematic error represents uncertainties in the treatment procedure or the system itself, and random error literally represents the random error that includes daily fluctuations in patient motion or internal organ motion^32^. Systematic error was calculated as the standard deviation of the average setup error of each patient throughout the whole fractions. Random error was calculated as the root mean square of the standard deviations in each patient over the whole patient group^33^. Intrafractional movements were evaluated on the post-treatment MVCT scan at each fraction. Moreover, the median values of displacement from post-treatment MVCT scans were calculated for whole fractions in each patient. Concerning the PTV margin at the L-S spine level, significant intrafractional displacement was defined as a displacement of > 10 mm in any axis in any fraction.

### Risk factors for intrafractional movement

Logistic regression analysis was used to identify risk factors for intrafractional movement. Statistically significant factors and clinically relevant factors (age, BMI, weight loss, rest during treatment, nausea, sedation during treatment, concurrent chemotherapy, total HT-CSI dose, total duration of HT-CSI, and beam on time) were determined via univariate and multivariate analyses. Patients’ body weight values determined on the first and last treatment days were used to calculate the rate of body weight change. Multivariate logistic regression analysis was then conducted using the backward stepwise selection procedure. All p values lower than 0.05 were considered statistically significant. Statistical analysis was performed using IBM SPSS, version 23.0 (IBM Corp., Armonk, NY, USA).

## Supplementary Information

Supplementary Information

## Data Availability

There are no restrictions on the availability of materials or information. The datasets generated during and/or analyzed during the current study are available from the corresponding author on reasonable request.

## References

[1] Mah K (1998). Computed tomographic simulation of craniospinal fields in pediatric patients: Improved treatment accuracy and patient comfort. Int. J. Radiat. Oncol. Biol. Phys..

[2] Michalski JM, Klein EE, Gerber R (2002). Method to plan, administer, and verify supine craniospinal irradiation. J. Appl. Clin. Med. Phys..

[3] Thomadsen B, Mehta M, Howard S, Das R (2003). Craniospinal treatment with the patient supine. Med. Dosim..

[4] Yoon M (2011). Craniospinal irradiation techniques: A dosimetric comparison of proton beams with standard and advanced photon radiotherapy. Int. J. Radiat. Oncol. Biol. Phys..

[5] Parker WA, Freeman CR (2006). A simple technique for craniospinal radiotherapy in the supine position. Radiother. Oncol..

[6] Seravalli E (2018). Dosimetric comparison of five different techniques for craniospinal irradiation across 15 European centers: analysis on behalf of the SIOP-E-BTG (radiotherapy working group). Acta Oncol..

[7] Tatcher M, Glicksman AS (1989). Field matching considerations in craniospinal irradiation. Int. J. Radiat. Oncol. Biol. Phys..

[8] Armstrong GT, Stovall M, Robison LL (2010). Long-term effects of radiation exposure among adult survivors of childhood cancer: Results from the childhood cancer survivor study. Radiat. Res..

[9] Chang EL (2002). Acute toxicity and treatment interruption related to electron and photon craniospinal irradiation in pediatric patients treated at the University of Texas M. D. Anderson Cancer Center. Int. J. Radiat. Oncol. Biol. Phys..

[10] St Clair WH (2004). Advantage of protons compared to conventional X-ray or IMRT in the treatment of a pediatric patient with medulloblastoma. Int. J. Radiat. Oncol. Biol. Phys..

[11] de Graaf SS (1996). Renal function after unilateral nephrectomy for Wilms' tumour: The influence of radiation therapy. Eur. J. Cancer.

[12] Mu X (2005). Does electron and proton therapy reduce the risk of radiation induced cancer after spinal irradiation for childhood medulloblastoma? A comparative treatment planning study. Acta Oncol..

[13] Paulino AC (2000). Late effects in children treated with radiation therapy for Wilms' tumor. Int. J. Radiat. Oncol. Biol. Phys..

[14] Schneider U, Zwahlen D, Ross D, Kaser-Hotz B (2005). Estimation of radiation-induced cancer from three-dimensional dose distributions: Concept of organ equivalent dose. Int. J. Radiat. Oncol. Biol. Phys..

[15] Mackie TR (1993). Tomotherapy: A new concept for the delivery of dynamic conformal radiotherapy. Med. Phys..

[16] Chung Y, Yoon HI, Kim JH, Nam KC, Koom WS (2013). Is helical tomotherapy accurate and safe enough for spine stereotactic body radiotherapy?. J. Cancer Res. Clin. Oncol..

[17] Chung Y, Yoon HI, Ha JS, Kim S, Lee IJ (2015). A feasibility study of a tilted head position in helical tomotherapy for fractionated stereotactic radiotherapy of intracranial malignancies. Technol. Cancer Res. Treat..

[18] Cozzi L, Clivio A, Vanetti E, Nicolini G, Fogliata A (2006). Comparative planning study for proton radiotherapy of benign brain tumors. Strahlenther. Onkol..

[19] Langner UW, Molloy JA, Gleason JF, Feddock JM (2013). A feasibility study using TomoDirect for craniospinal irradiation. J. Appl. Clin. Med. Phys..

[20] Mizumoto M, Oshiro Y, Yamamoto T, Kohzuki H, Sakurai H (2017). Proton beam therapy for pediatric brain tumor. Neurol. Med. Chir. (Tokyo).

[21] Pai Panandiker A (2007). Craniospinal irradiation with spinal IMRT to improve target homogeneity. Int. J. Radiat. Oncol. Biol. Phys..

[22] Penagaricano JA, Papanikolaou N, Yan Y, Youssef E, Ratanatharathorn V (2005). Feasibility of cranio-spinal axis radiation with the Hi-Art tomotherapy system. Radiother. Oncol..

[23] Penagaricano JA, Yan Y, Corry P, Moros E, Ratanatharathorn V (2007). Retrospective evaluation of pediatric cranio-spinal axis irradiation plans with the Hi-ART tomotherapy system. Technol. Cancer Res. Treat..

[24] Schiopu SR (2017). Craniospinal irradiation using helical tomotherapy for central nervous system tumors. J. Radiat. Res..

[25] Penagaricano J, Moros E, Corry P, Saylors R, Ratanatharathorn V (2009). Pediatric craniospinal axis irradiation with helical tomotherapy: Patient outcome and lack of acute pulmonary toxicity. Int. J. Radiat. Oncol. Biol. Phys..

[26] Sterzing F (2008). Helical tomotherapy. Experiences of the first 150 patients in Heidelberg. Strahlenther. Onkol..

[27] Gupta T (2015). Assessment of three-dimensional set-up errors using megavoltage computed tomography (MVCT) during image-guided intensity-modulated radiation therapy (IMRT) for craniospinal irradiation (CSI) on helical tomotherapy (HT). Technol. Cancer Res. Treat..

[28] Tasson A, Laack NN, Beltran C (2018). Clinical implementation of robust optimization for craniospinal irradiation. Cancers.

[29] Forrest LJ (2004). The utility of megavoltage computed tomography images from a helical tomotherapy system for setup verification purposes. Int. J. Radiat. Oncol. Biol. Phys..

[30] Meeks SL (2005). Performance characterization of megavoltage computed tomography imaging on a helical tomotherapy unit. Med. Phys..

[31] Organization, W. H. *Obesity: Preventing and Managing the Global Epidemic*. (World Health Organization, 2000). 11234459

[32] Thondykandy BA (2015). Setup error analysis in helical tomotherapy based image-guided radiation therapy treatments. J. Med. Phys..

[33] Al-Wassia R, Bahig H, Poon E, Parker W, Freeman C (2013). Daily setup uncertainty analysis for craniospinal irradiation using helical tomotherapy. Pract. Radiat. Oncol..

[34] Mongioj V (2011). Set-up errors analyses in IMRT treatments for nasopharyngeal carcinoma to evaluate time trends, PTV and PRV margins. Acta Oncol..

[35] Hou W-H, Wang C-W, Tsai C-L, Hsu F-M, Cheng JC-H (2016). The ratio of weight loss to planning target volume significantly impacts setup errors in nasopharyngeal cancer patients undergoing helical tomotherapy with daily megavoltage computed tomography. Radiol. Oncol..

