# Supplementary material for: Practical aspects of the application of helical tomotherapy for craniospinal irradiation
Source: Sci Rep. 2021 Mar 17;11:6120. doi: 10.1038/s41598-021-85574-y (PMC7969733; doi:10.1038/s41598-021-85574-y)
Supplement: Supplementary file 1 — Supplementary Information [file 41598_2021_85574_MOESM1_ESM.docx]

**Supplement Table S1.** Comparison of patient and treatment characteristics between pediatric and adult

| Characteristic | Pediatric | | Adult | |
| --- | --- | --- | --- | --- |
|  | N = 40 | *%* | N = 43 | *%* |
| Age (years, median [range]) | 11.5 (2-19) | | 46 (20-74) | |
| Sex |  |  |  |  |
| Male | 26 | 65.0 | 28 | 65.1 |
| Female | 14 | 35.0 | 15 | 34.9 |
| Height (cm, median [range]) | 144 (88-192) | | 170 (146-180) | |
| Body-mass index (kg/m², median [range]) | 18.9 (10.9-28.4) | | 21.2 (14.5-26.4) | |
| Total CSI dose (Gy, median [range]) | 23.4 (12.0-45.0) | | 36.0 (12.0-45.0) | |
| Total CSI fraction number (fractions, median [range]) | 13 (8-27) | | 24 (10-30) | |
| Fractional CSI dose (Gy, median [range]) | 1.5 (1.5-2.0) | | 1.5 (1.2-3.0) | |
| CSI field |  |  |  |  |
| Brain-Sacrum | 40 | 100.0 | 36 | 83.7 |
| Posterior fossa-Sacrum | 0 | 0.0 | 2 | 4.7 |
| C1 spine-Sacrum | 0 | 0.0 | 5 | 11.6 |
| Beam on time (seconds, median [range]) | 517.4 (317.4-964.8) | | 614.3 (310.6-924.6) | |
| Sedation during treatment | 5 | 12.5 | 2 | 4.7 |
| Concurrent chemotherapy | 18 | 45.0 | 11 | 25.6 |
| Overall CSI treatment time (days, median [range]) | 24 (11-81) | | 35 (12-108) | |
| Medically-indicated treatment interruptions | 14 | 35.0 | 14 | 32.6 |
| Days (median [range]) | 5 (1-38) | | 6 (1-58) | |
| Adaptive during CSI | 0 | 0.0 | 3 | 7.0 |
| Weight loss grade ≥ 1* | 7 | 17.5 | 6 | 14.0 |
| Nausea grade ≥ 2* | 11 | 27.5 | 9 | 20.9 |

***Abbreviations*:** CSI, craniospinal irradiation; Gy, gray
* Based on Common Terminology Criteria for Adverse Events version 5.0
